# Application of image processing and soft computing strategies for non-destructive estimation of plum leaf area

**DOI:** 10.1371/journal.pone.0271201

**Published:** 2022-07-11

**Authors:** Atefeh Sabouri, Adel Bakhshipour, MohammadHossein Poornoori, Abouzar Abouzari

**Affiliations:** 1 Department of Agronomy and Plant Breeding, Faculty of Agricultural Sciences, University of Guilan, Rasht, Iran; 2 Department of Biosystems Engineering, Faculty of Agricultural Sciences, University of Guilan, Rasht, Iran; 3 Crop and Horticultural Science Research Department, Mazandaran Agricultural Resources Research and Education Center, Agricultural Research, Education and Extension Organization (AREEO), Tehran, Iran; Universidade Federal de Uberlandia, BRAZIL

## Abstract

Plant leaf area (LA) is a key metric in plant monitoring programs. Machine learning methods were used in this study to estimate the LA of four plum genotypes, including three greengage genotypes (*Prunus domestica* [subsp. *italica* var. *claudiana*.]) and a single myrobalan plum (*prunus ceracifera*), using leaf length (L) and width (W) values. To develop reliable models, 5548 leaves were subjected to experiments in two different years, 2019 and 2021. Image processing technique was used to extract dimensional leaf features, which were then fed into Linear Multivariate Regression (LMR), Support Vector Regression (SVR), Artificial Neural Networks (ANN), and the Adaptive Neuro-Fuzzy Inference System (ANFIS). Model evaluation on 2019 data revealed that the LMR structure LA = 0.007+0.687 L×W was the most accurate among the various LMR structures, with R^2^ = 0.9955 and Root Mean Squared Error (RMSE) = 0.404. In this case, the linear kernel-based SVR yielded an R^2^ of 0.9955 and an RMSE of 0.4871. The ANN (R^2^ = 0.9969; RMSE = 0.3420) and ANFIS (R^2^ = 0.9971; RMSE = 0.3240) models demonstrated greater accuracy than the LMR and SVR models. Evaluating the models mentioned above on data from various genotypes in 2021 proved their applicability for estimating LA with high accuracy in subsequent years. In another research segment, LA prediction models were developed using data from 2021, and evaluations demonstrated the superior performance of ANN and ANFIS compared to LMR and SVR models. ANFIS, ANN, LMR, and SVR exhibited R^2^ values of 0.9971, 0.9969, 0.9950, and 0.9948, respectively. It was concluded that by combining image analysis and modeling through ANFIS, a highly accurate smart non-destructive LA measurement system could be developed.

## Introduction

European nations introduced the first plum varieties, including damson (*Prunus domestica subsp*. *insititia*) and greengage (*Prunus domestica subsp*. *italica var*. *claudiana*.). Greengages are indigenous to continental Europe [1]. Plums, which are members of the *Prunus genus*, in production among the stone fruits are in second place after peaches and nectarines. Due to the genus’s diversity center’s proximity to the origin of the first human civilizations, it was one of the first fruits considered by humans [2]. However, the genus’s antiquity dating back to Roman times [3] and natural intraspecific crosses may account for the existence of more than 2,000 varieties, although only a few of them are commercially valuable [4, 5]. Plums are effective in the prevention and treatment of many diseases due to the presence of beneficial compounds to human health such as antioxidant and phenolic compounds, dietary fiber, isatin, lutein, cryptoxanthin, zeaxanthin sorbitol, potassium, fluoride, iron, and vitamins A, C, and B-complex groups such as vitamin B-6, pantothenic acid, and niacin [4].

Iran’s indigenous wild fruit trees are poorly documented, with only a few cases reported [3]. Cherry plum (*Prunus divaricata* Ledeb.) is a diploid wild plum species that grows to a height of 10 meters as a thornless deciduous shrub or tree and is self-incompatible with edible fruits. The Balkan Peninsula, Central Europe, the Caucasus, and Central Asia, including northern Iran, are native to this species [6]. Various non-wood forest products and forest fruits, including *Prunus divaricata* Ledeb, contribute significantly to the economic well-being of some rural households in northern Iran’s forests, both directly and indirectly. Due to its extensive use as spring fruits and as a rootstock, this wild species increases resistance to root-knot nematodes in peach and plum nurseries, making it an ideal candidate for further study and domestication [5].

Determining the effective parameters affecting plant yield is a goal shared by many crops and horticulture researchers. Meanwhile, measuring the leaf area (LA) plays a vital role in evaluating plant growth and development in a variety of ecophysiology and physiology studies [7–9]. LA is an essential structural feature of plants and has long been a focus of research in plant science. It is the main level of exchange of matter and energy between the tree canopy and the atmosphere and is associated with various processes such as light absorption, evaporation, transpiration, and photosynthesis [9, 10].

Accurate LA measurement is critical for plants whose fruit size is economically valuable, as fruit load and LA are correlated [9, 11]. Additionally, it is essential to evaluate tree pruning systems and determine the pest population density [12]. LA and its derived parameters, such as leaf area index (LAI), net assimilation rate (NAR), specific leaf area (SLA), specific leaf weight (SLW), and leaf area duration (LAD), are significant for evaluating the relationship between plants and their environment [7, 8]. Because LA and yield are related, evaluating LA can aid in identifying superior genotypes, particularly under abiotic stress conditions. LA has been demonstrated to be reduced by drought, and drought-resistant Jerusalem artichoke genotypes could retain LA [13]. The LA was identified as a significant morpho-physiological trait in the screening of cowpea (*Vigna unguiculata* (L.) Walp.) genotypes for waterlogging tolerance [14]. Sravanthi and Ratnakumar [15] demonstrated that the superior sesame (*Sesamum indicum* L.) genotype IC-204966 for LA was significantly different from other genotypes under deficit moisture stress.

Regarding the physiological and ecological significance of leaf area, accurate measurement of LA is necessary for elucidating the interaction between plant growth and the environment. Today, mathematical models used to predict the growth of plant components provide reliable criteria. Numerous methods have been developed to facilitate LA measurement. These factors can be determined destructively or non-destructively [16, 17]. Harvesting leaves is required for conventional and destructive methods, which is costly and a time-consuming and challenging process. On the other hand, these methods may indicate the impossibility of studying leaf growth trends throughout the growing season, thereby lowering the accuracy of assessments. Developing LA estimation models that are adjusted for leaf L and W enables a non-destructive, precise, and rapid evaluation process and the ability to track leaf growth accurately and monitor plant health more effectively.

Artificial neural network (ANN) is one of the most widely used and reliable approaches in various sectors of the food and agricultural industries. The ANN algorithm is a supervised machine learning algorithm that simulates the human brain’s classification function for regression-type applications [18, 19]. An ANN structure consists of an input layer, an output layer, one or more processing layers referred to as hidden layer(s), and a collection of processing elements referred to as neurons. Each neuron’s weight and bias values are adjusted throughout the training process to minimize errors and optimize classification or prediction accuracy until the predefined performance conditions are satisfied [20]. ANNs have been used in a variety of agricultural studies, including the prediction of plant biology processes [21], the prediction of genetic merit for flowering traits [22], the identification of plants and weeds [23, 24], and yield prediction [25, 26]. Moreover, there are some excellent review articles on the applications of ANNs in agriculture [27–29].

Kumar et al. [11] utilized the ANN model to estimate durian LA based on leaf L and W. The best-fitting result was obtained by an ANN with one hidden layer and two neurons in the hidden layer. The values of coefficient of determination. The R^2^ and RMSE of this model were 0.94 and 4.81 on test data, respectively. Another study used an ANN with a single hidden layer of six neurons to estimate the LA of the invasive Wedelia plant, with R^2^ and RMSE values of 0.96 and 0.379, respectively [30]. Furthermore, several other publications report on the successful use of ANN in estimating plant LA [31–35].

Adaptive Neuro-Fuzzy Inference System (ANFIS) is a more recent intelligent supervised hybrid machine learning technique that solves classification and modeling problems by combining the learning capabilities of ANNs and fuzzy logic systems [36]. Shastry and Sanjay [37] describe the fundamental concept and architecture of ANFIS, as well as some of the applications of ANFIS in agriculture. Sabouri and Sajadi [38] demonstrated the efficacy of using ANFIS and ANN modeling to predict the LA of bread wheat, durum wheat, and triticale plants using image-extracted L and W dimension values. Additionally, ANFIS was successfully used to predict LA plant species using leaf L, leaf W, plant type, and a specific coefficient defined for each plant with an R^2^ = 0.997.

Support Vector Regression (SVR) is a generalization of Support Vector Machine (SVM) that incorporates regression functions into SVM to solve regression problems [39, 40]. As a supervised machine learning algorithm, SVR has a high capability in regression modeling [41, 42]. SVR is a kernel-based technique in which the kernel function projects the input data into higher-dimensional feature space to find the hyperplane with the lowest error margin and the best fit to the regression line [43, 44]. A comparison study conducted by Abdel-Sattar and Aboukarima [45] proved the superiority of ANN and SVR methods over linear regression methods (LRM) for predicting the mass of Indian jujube fruits based on their axial dimensions. Sabouri and Sajadi [46] recently reported that using ANFIS and SVR methods, they were able to predict the LA of chia (*Salvia hispanica* L.) and quinoa (*Chenopodium quinoa* Willd.) with high accuracy (R^2^ > 98%), while demonstrating that methods based on artificial intelligence are capable of accurately estimating a plant’s LA.

To our knowledge, there is no study comparing the capability of soft computing methods for the estimation of plum LA. The main contribution of this work was to introduce a model to be used for the non-destructive measurement of plum leaf area based on its length and width values. To reach this aim we collected a big database that can also be used by researchers for their further works. Thus, this study aimed to investigate the LA measurement applications of LRM, ANN, ANFIS, and SVR algorithms. To this end, various modeling strategies were compared, and the most successful plum LA estimator was introduced. Furthermore, the approaches mentioned above’ generalizability were investigated to develop a comprehensive tool for estimating the LA of multiple plum genotypes using a single universal model.

## Materials and methods

### Data collection

**Fig 1:**
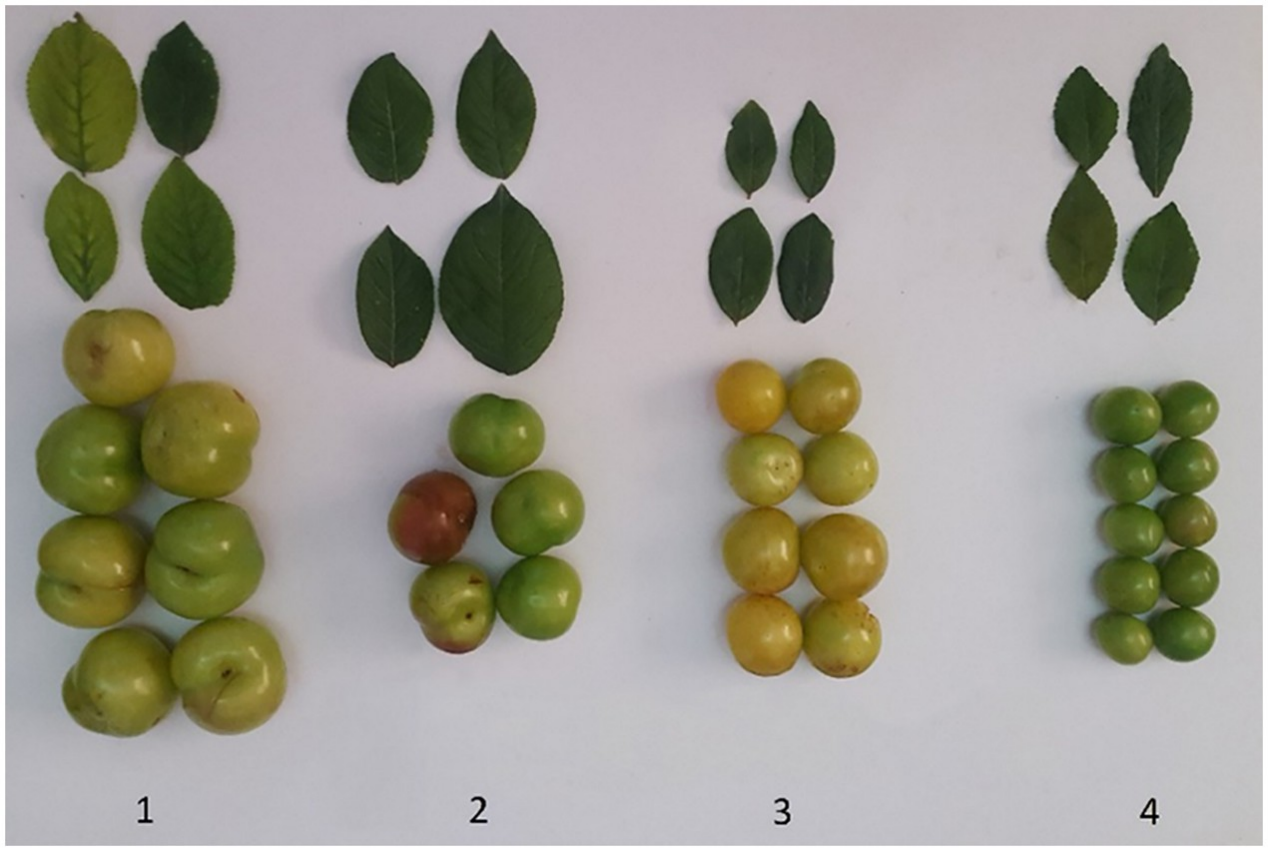
The leaves and fruits of three greengage genotypes [*Prunus domestica* (subsp. *italica* var. *claudiana*.)] with local names Gavali (1), Ghandi (2) and Shahryari (3) and a myrobalan (4) plum (prunus ceracifera) with local name of Jangali.

Three greengage genotypes [*Prunus domestica* (subsp. *italica* var. *claudiana*.)] with local names Gavali, Ghandi and Shahryari and a myrobalan plum (*prunus ceracifera*) with the local name of Jangali were used to develop and validate the LA prediction models. The age of the trees varied between 15 and 26 years. Leaf samples were picked carefully and placed in a cooler and immediately were transported to the laboratory. These genotypes were determined as an example of greengage and myrobalan plums throughout the northern region of Iran. Images of the studied plums are presented in Fig 1. The fruits are also included in this figure to help better understanding of readers about these genotypes.

Data collection was performed in two series. The first experiment was carried out during the 2019 growing seasons by collecting 1224 undamaged leaves at all stages of development of Gavali greengage randomly from different levels of the canopy in the four directions of the crown from the research orchard of the Faculty of Agriculture, the University of Guilan, Rasht, Iran (37°16_N, 51°3_E). The second data collection phase was done in 2021, in which all of the four mentioned genotypes were experimented. The numbers of collected data for each genotype are available in Table 1. It is also worth noting that data validation was performed before data analysis and removed all data that were outliers or unacceptable.

**Table 1:** Performance values of image processing technique for measuring the leaf shape features.

Shape feature	R^2^	RMSE
Leaf length	0.9999	0.0100
Leaf width	0.9999	0.0084
Leaf Area	0.9999	0.0225

To accelerate the process of extracting the required information from a large number of leaf samples, the image analysis method was used. In order to acquire the desired images, the collected leaf samples were placed on a mate white platform and the images were captured using a smartphone which was placed perpendicularly above the samples with a constant vertical distance of 55 cm. The acquired images having the frame size of 5312 × 2988 pixels, were transferred to the computer for further processing.

### Image processing and feature extraction

**Fig 2:**
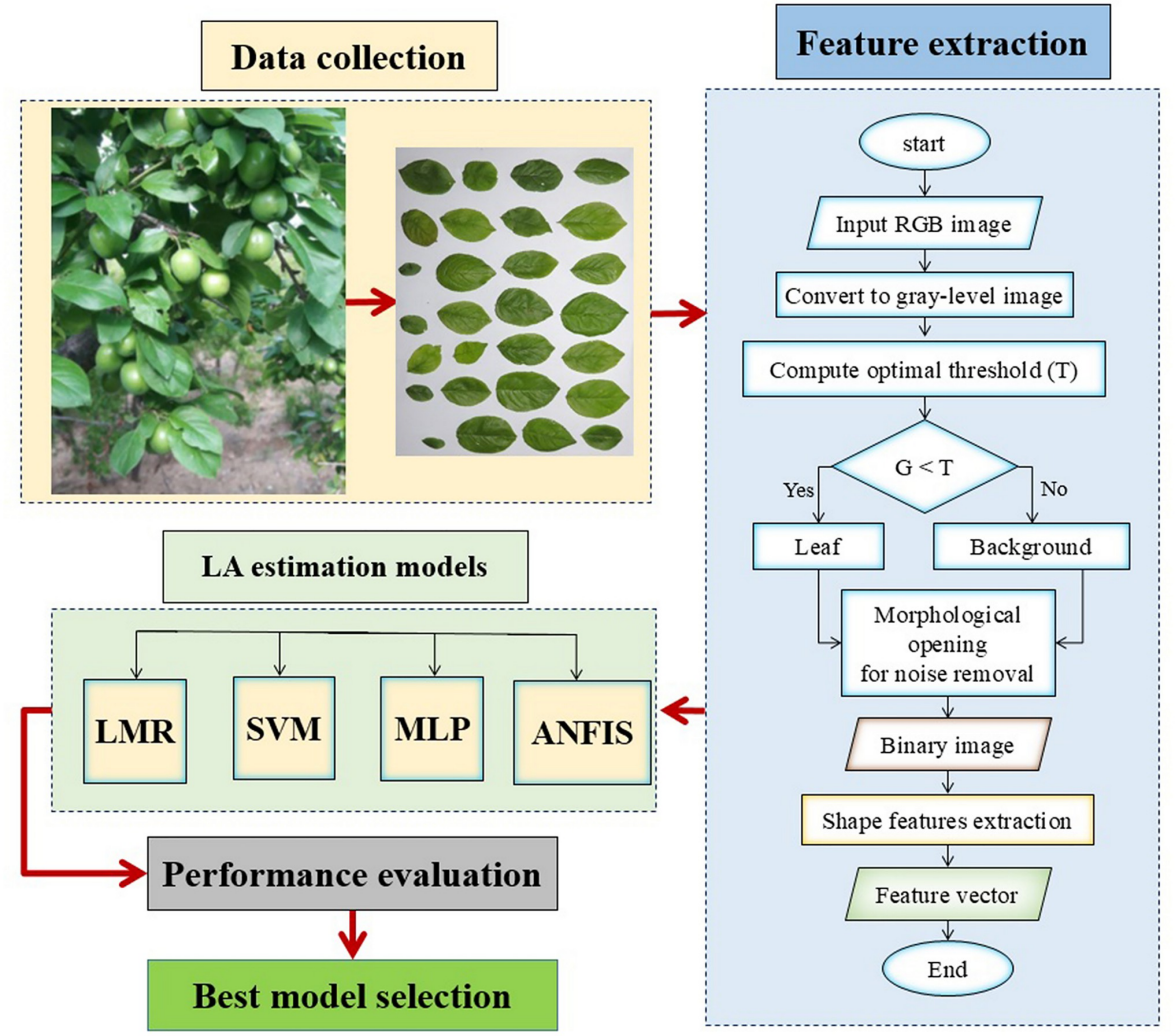
Methodology diagram of the LA estimation model development.

Fig 2 shows the diagram of the methodology used in this study for LA estimation model development. To extract L, W, and LA values of the sample leaves, the RGB images of leaves were loaded into and processed in the image processing toolbox of MATLAB programming software (The MathWorks, USA, version R2018b). The primary images were in RGB space, therefor the red (R), green (G), and blue (B) color layers were extracted. Regarding the green color of the plum leaves, the popular Excessive Green index (EGI) method was used to identify leaf regions in images. The ExG component was calculated using Eq 1 [47]:

ExG=2g-r-b (1)

where the r, g, and b values were extracted using Eqs 2 to 4 [47]:

$$r=\frac{R}{R+G+B}$$ (2)

$$g=\frac{G}{R+G+B}$$ (3)

$$b=\frac{B}{R+G+B}$$ (4)

Otsu optimal thresholding method [48] was applied on the ExG image to segment leaf regions from the image background. The attained binary image contained the leaves regions in white (with 1 value), as regions of interest (ROI), the image background in black (with zero value), and also having some possible unwanted white points. Thereupon, morphological opening (an erosion followed by a dilation) was applied using “*imopen*” function in the MATLAB programming software, to remove these noises. Regions of different leaves in the resulting black-and-white (binary) image were labeled and the leaf L, W and LA values were extracted from the leaf binary images.

Image of a white square with known 1 cm ×1 cm dimensions was captured in a similar condition to leaves and used to convert the pixel-based extracted values of leaves to centimeters.

### Regression models

Linear regression analysis was performed to build the best model for predicting LA by using the Excel 2016 software (Microsoft Corporation, Redmond, WA). With considering different subsets of leaf data, including W, L, W^2^, L^2^, L+W, product (L×W), (L+W)^2^, and the square root of (L×W)^2^, as independent variables, and LA as the dependent variable, regression modeling was conducted.

### ANN

In this study, the well-known Multi-Layer Perceptron (MLP) neural network with one hidden layer was developed in MATLAB programming software to estimate the LA based on L and W values. Error backpropagation algorithm was used to adjust the weights and biases of networks. Different MLPs were developed and compared by changing the number of neurons in the hidden layer (1 neuron to 30 neurons), the type of transfer function (Tangent sigmoid (TS) and logarithmic sigmoid (LS)), and the training technique (Levenberg-Marquardt (LM), Scaled conjugate gradient (SCG), and Bayesian regularization (BR)). The transfer function of the output layer was pure-line. Values of leaf L and W were fed into the MLPs as input data and the LA values were the target data. The dataset was divided randomly into training data (60%), cross-validation data (20%) and model test data (20%).

### ANFIS

Sugeno-based ANFIS models were developed in MATLAB programming environment using grid partitioning technique for the estimation of plum LA based on leaf L and W values. Different types of input Membership Functions (Gaussian, Sigmoidal, and Triangular), output Membership Functions (linear and constant MFs), and Optimization Methods (Back-Propagation and the hybrid), besides different number of Membership Functions (2, 3, and 4 Membership Function for each input variable) were evaluated to find the most accurate ANFIS estimator model. The dataset segmentation for ANFS training, cross-validation, and test was performed such as that described previously for the ANN model.

### SVR

The foundational parameters of SVR algorithms are kernels. The accuracy of SVR models having different kernels, including Linear, Polynomials (2 and 3 dimensional), RBF, and Sigmoid were evaluated to find the most efficient SVR algorithm for LA estimation.

### Evaluation of developed models

In order to find the most suitable LA estimator engines, the developed models were selected by simultaneously considering the highest coefficient of determination (R^2^) and the least RMSE [49]. These criteria were calculated using Eqs 5 and 6:

$$R^{2}=1-\left[\frac{\sum_{i=1}^{n}{\left({LA}_{mea,i}-{LA}_{est,i}\right)}^{2}}{\sum_{i=1}^{n}{({LA}_{mea,i}-\overset{-}{{LA}_{mea}})\;}^{2}}\right]$$ (5)

$$RMSE={\left[\frac{1}{n}\sum_{i=1}^{n}{\left({LA}_{mea,i}-{LA}_{est,i}\right)}^{2}\right]}^{0.5}$$ (6)

where, LA_*est*,*i*_, LA_*mea*,*i*_, $\overset{-}{{LA}_{mea}}$, n and k, are the *i*th estimated value, the *i*the measured value, the average of observed values, the total number of LA data, and the number of model parameters, respectively.

### Model validation

A proposed model is valuable if it can be applied with high reliability in next experiments even in future years. In this regard, the models developed by the data of 2019, were validated using the data of the 2021 experiments on four genotypes. One genotype was the same as that was used for developing the model (greengage Gavali) and three more genotypes including Ghandi and Shahryari genotypes from greengage (*prunus domestica*) and Jangali genotype from plum myrobalan (*prunus ceracifera*). Leaf W, L, and actual LA values were measured for 1470 leaves of Gavali genotype, 899 leaves of Shahryari genotype, 925 leaves of Ghandi genotype, and 1030 leaves of Jangali genotype, respectively. The L and actual LA of 2021 samples were fed into pre-developed models. The R^2^ and RMSE values between actual 2021 LA values and the corresponding estimated values were calculated to show the effectiveness of trained LRM, ANN, ANFIS, and SVR models.

## Results

### Image processing

**Fig 3:**
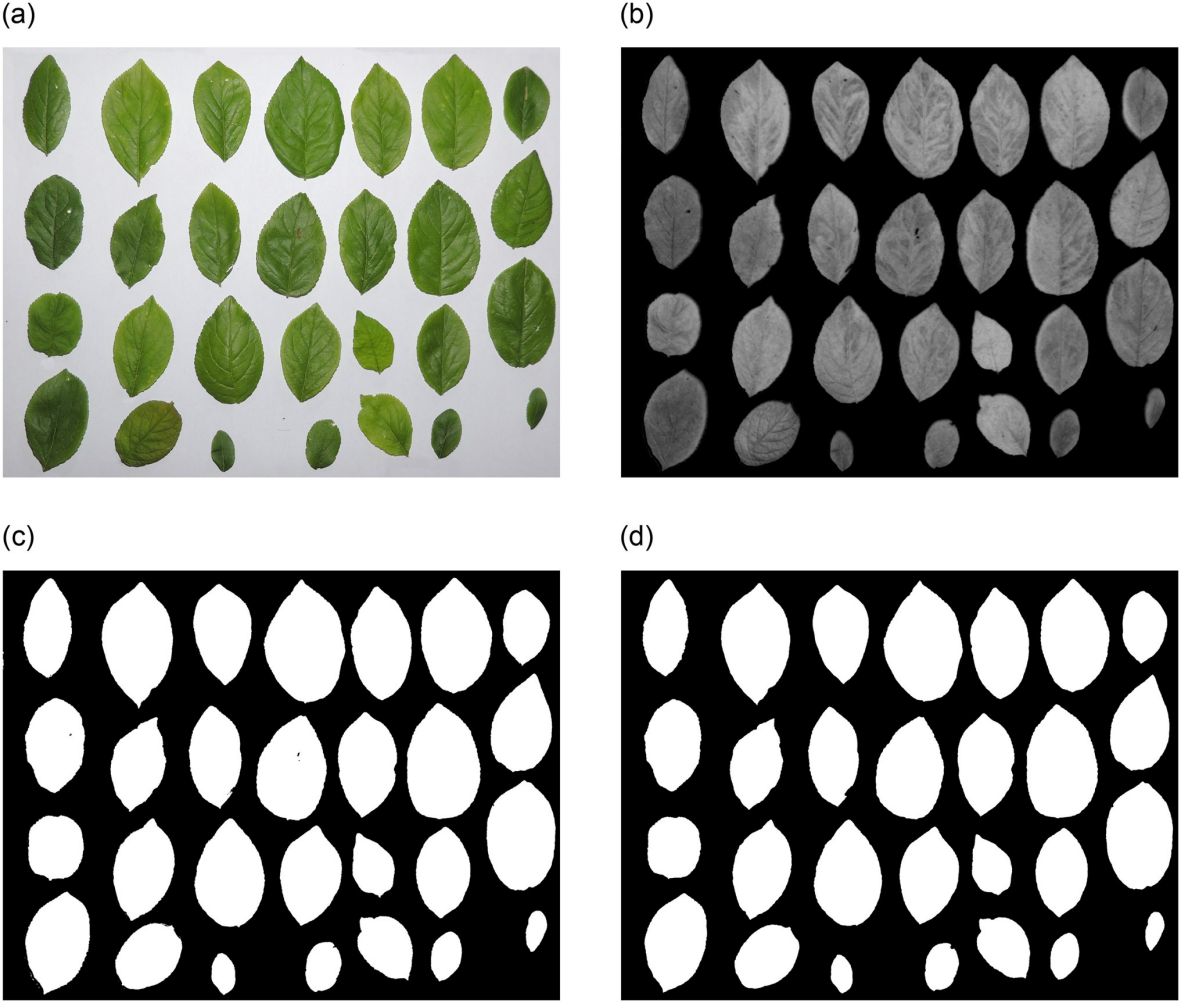
Results of image segmentation steps; a) primary RGB image, b) EXG image, c) first obtained binary image after implementing optimal threshold on the EXG image, and d) final binary image after removing small objects (noises) and filling inside leaf regions.

A gallery of the results of different image segmentation steps is provided in Fig 3. It can be seen that all the leaves were completely separated from the image background to be then used for dimension extraction.

In order to provide a reliable application of image analysis for plum leaf characteristics, a number of 100 leaves were selected randomly and the image extracted L and W values were compared to the manual measurements.

The evaluation results are presented in Table 1 which shows that the image processing method has a very high ratio of performance in leaf characteristic measurement. The R^2^ values of more than 0.9999 show the very high degree of accuracy and applicability of the image processing technique for the measurement of leaf shape characteristics. So this method was used to extract leaf data to be used for model development operations.

### Descriptive statistics of collected data from leaves

Descriptive statistics of three leaf morphology measures including L, W, and LA values are presented in Table 2 for three greengage genotypes and one myrobalan plum based on collected data in 2019 and 2021.

**Table 2:** Descriptive statistics of leaf Length (L), width (W) and area (LA) of three greengage genotypes with local names Gavali, Ghandi and Shahryari and a myrobalan plum with the local name of Jangali.

Genotype name	year	Population size	Leaf L (cm)			Leaf W (cm)			LA (cm)^2^		
			Mean±SE	Min	Max	Mean±SE	Min	Max	Mean±SE	Min	Max
Gavali	2019	1224	4.5753±0.0400	1.3045	9.1903	2.6192±0.0284	0.8485	6.4840	9.1375±0.1725	1.0271	39.8978
Gavali	2021	1470	4.2248±0.0287	0.9129	20.6068	2.3995±0.0170	0.5052	4.4509	7.5005±0.0966	0.3952	20.9407
Shahryari	2021	899	3.5501±0.0450	0.9562	7.6593	1.7086±0.0271	0.4664	4.8096	4.8274±0.1403	0.3246	25.1487
Ghandi	2021	925	2.9602±0.0334	0.9267	8.1396	1.4453±0.0201	0.4195	4.2884	3.2748±0.0798	0.3106	20.9286
Jangali	2021	1030	2.5951±0.0279	0.9906	6.4329	1.2001±0.0147	0.4989	3.3662	2.3161±0.0516	0.3549	11.9520

### LRM model

#### Fitting LRMs on 2019 data to develop the model

Different mathematical equations for estimating the LA of plum (Accession 1) in 2019 are shown in Table 3. The Independent variable L and W product (L×W) was identified as the best model, giving the lowest RMSE (0.4037) value, also the highest R^2^ (0.9955).

**Table 3:** Fitted models to estimate the LA from leaf length (L) and leaf width (W) in plum (*prunus vachuschtii*, Alucheh Gavali) in 2019.

	Model	A	B	R^2^	RMSE
1	LA = a+b(L×W)	0.0074	0.6871	0.9955	0.4037
2	LA = a+b(L+W)^2^	-0.2942	0.1646	0.9912	0.5648
3	LA = a+b(W)^2^	1.2158	1.0100	0.9757	0.9406
4	LA = a+bW	-6.4560	5.9535	0.9578	1.2401
5	LA = a+b√(L×W)	-8.3383	5.0598	0.9566	1.2569
6	LA = a+b(L+W)	-8.7924	2.4922	0.9478	1.3792
7	LA = a+b(L)^2^	-0.8958	0.4383	0.9464	1.3973
8	LA = a+bL	-9.5096	4.0756	0.8942	1.9624

The model which was regressed independent variable only leaf length (L) had the lowest R^2^ value and the highest RMSE value, resulting it is less acceptable to plum LA estimation. While the use of only the leaf width in regression modeling of LA estimation appeared more successful.

**Fig 4:**
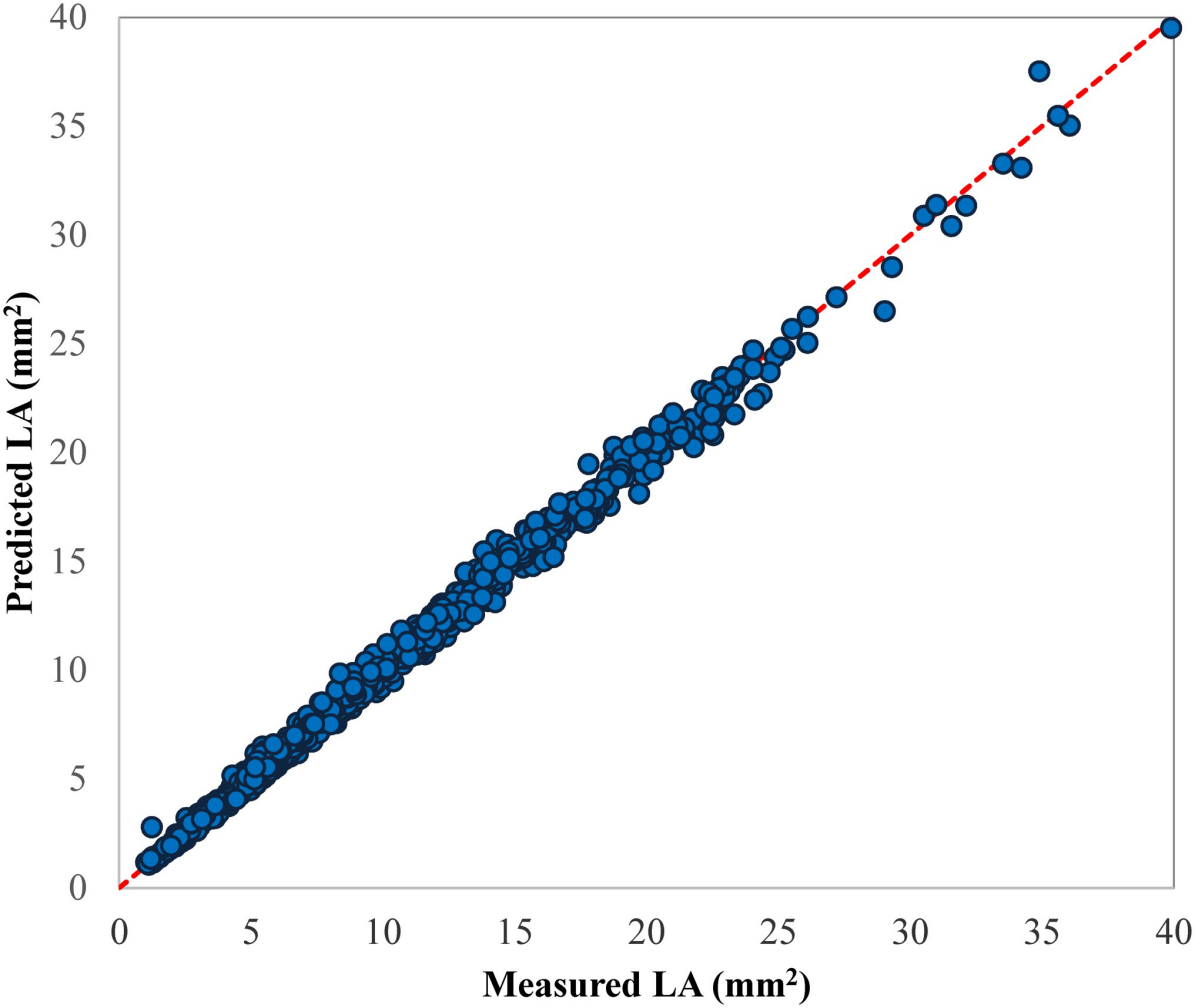
Scatter plot of the estimated LA values by LA = 0.007 + 0.687 L×W model vs. the corresponding measured LA values of 2019 dataset.

Based on selection criteria including lowest RMSE and highest R^2^, the best equation including product of L and W (LA = 0.007 + 0.687 L×W) was selected to accurately predict the plum LA. Fig 4 shows the predicted values by this model vs. the LA values measured in 2019.

#### Evaluation of the 2019 data-based LRM model with 2021 data

Data of Gavali accession and two other greengage accession from prunus cerasifera and one myrobalan plum cultivar (prunus cerasifera) in 2021 were used for validation of the selected model. In this regard, about 1000 leaves of four genotypes were taken during 2021. The W, L and actual LA were measured and LA predicted using the selected model. The estimated regression parameters, R^2^ and goodness of fit test between modeled and observed LA in four genotypes are shown in Table 4.

**Table 4:** Validation of “LA = 0.6871LW + 0.0074” model for estimation of LA of three greengage genotypes with local names Gavali, Ghandi and Shahryari, and a myrobalan plum with the local name of Jangali, recorded in 2021.

Genotype name	Botanical classified	Intercept (SE)	Coefficient regression (SE)	R^2^	RMSE
Gavali	*prunus domestica*	0.0137(0.0188)	1.0155(0.0229)	0.9926	0.3485
Shahryari	*prunus domestica*	-0.0289(0.0374)	0.9901(0.0420)	0.9971	0.2441
Ghandi	*prunus domestica*	0.0799(0.0415)	0.956(0.0325)	0.9936	0.2336
Jangali	*prunus cerasifera*	0.068(0.01287)	0.9312(0.0279)	0.9941	0.2017

**Fig 5:**
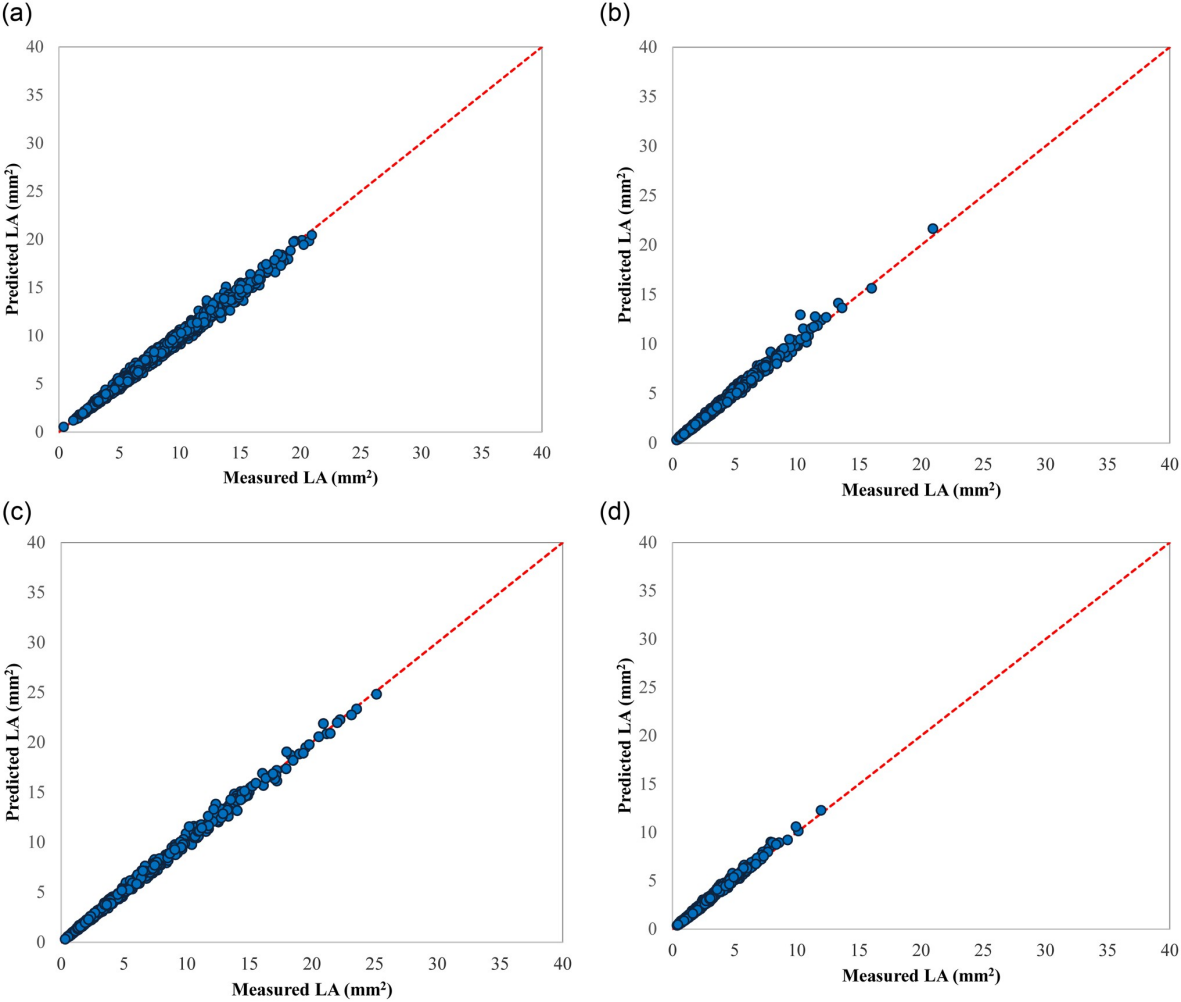
Scatter plots of the estimated LA values by LA = 0.007 + 0.687 L×W model (developed by 2019 data) vs. the corresponding measured LA values of 2021 dataset for different genotypes; a) Gavali, b) Ghandi, c) Shahryari, and d) Jangali.

Interestingly, the selected model in 2019 was not only able to predict LA for the same plum accession, but also succeeded in predicting the LA in large size populations for the other three genotypes. The coefficient of determination fitted regression between measured LA and predicted LA were more than 0.99 for four genotypes (Table 4). Moreover, the regression lines of predicted versus measured LA data were not significantly different from the slope (= 1) and intercept (= 0) 1:1 line (Fig 5a to 5d). Axis bounds are selected the same for all scatter plots to help better comparison.

#### Calibration of a universal model for LA estimation for plum by pooling data

We calibrated a LA estimation model derived from each genotype using collected data in 2021. Table 5 shows the results of regression analysis for four genotypes. According to the results, regression coefficients of four equations were not significantly different (Table 5). In addition, distribution of predicted LA data using the selected model derived from a single accession for all four genotypes were homogenous with their actual LA data (Table 5). Therefore, these results suggest that there is possible to calibrate a universal model for estimation of plum LA by pooling all data of four genotypes in 2021.

**Table 5:** The results of regression analysis for three greengages (*prunus domestica*) and a myrobalan plum (*prunus cerasifera*) genotypes to estimate the LA using the product of leaf length (L) and width (W) as independent variable [LA = a+b(L×W)] using collected data in 2021.

Genotype name	Botanical classified	a	b	R^2^	RMSE
Gavali	*prunus domestica*	0.0208	0.6977	0.9925	0.3194
Shahryari	*prunus domestica*	-0.0220	0.6802	0.9971	0.2277
Ghandi	*prunus domestica*	0.0886	0.6568	0.9936	0.1944
Jangali	*prunus cerasifera*	0.0745	0.6398	0.9941	0.1277

**Fig 6:**
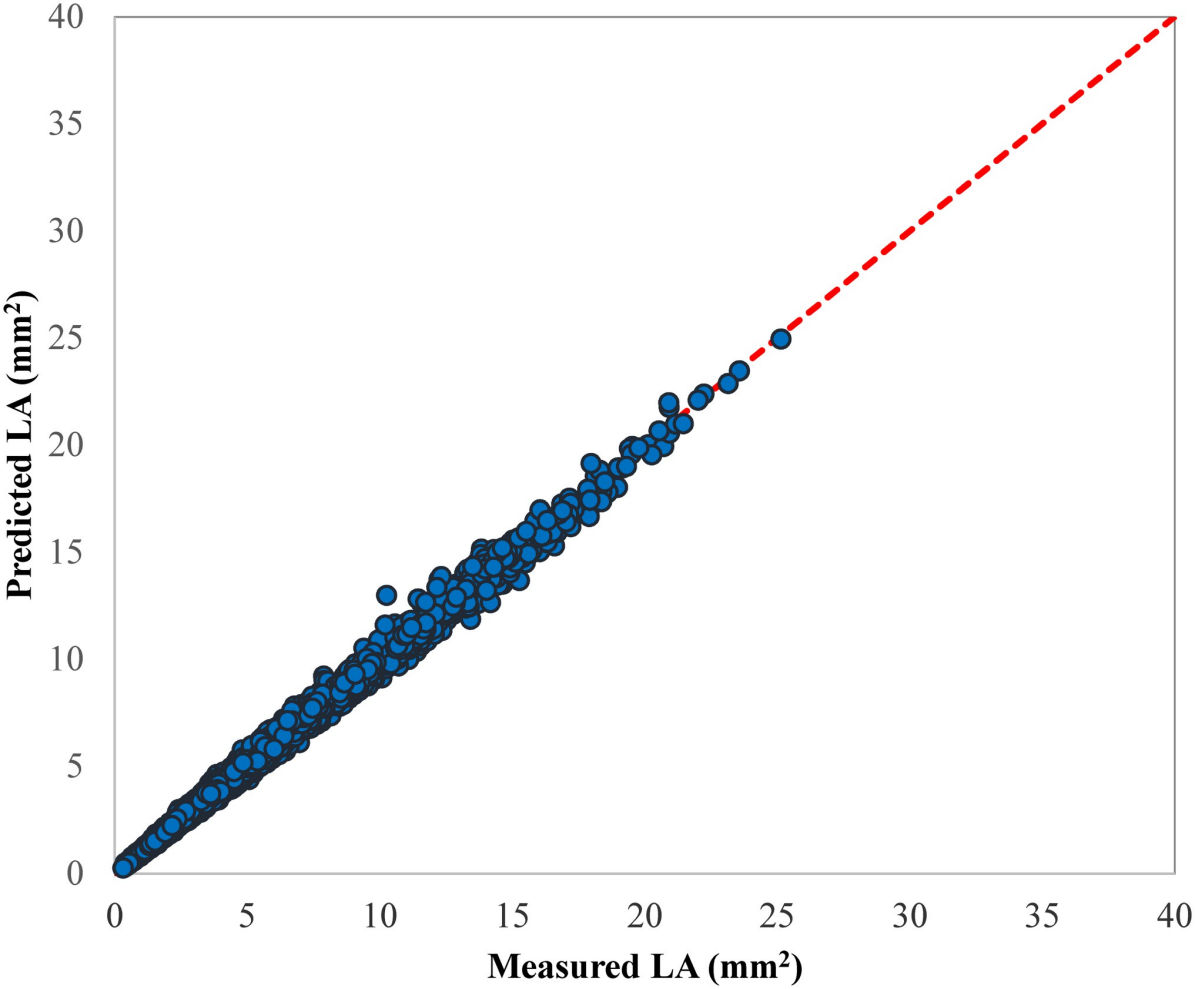
Scatter plot of the estimated LA values by universal regression model (LA = -0.039+0.6922 L×W) vs. the corresponding measured LA values of 2021 dataset containing all studied genotypes.

After approving those linear models derived from four genotypes for estimating LA are not significantly different, all 2021 data were pooled and a comprehensive model was developed to estimate the LA using product of L and W as the independent variables [LA = a+b(L×W)]. The resulting universal equation of the estimation model was LA = -0.039+0.6922 L×W (R^2^ = 0.9950, RMSE = 0.2723). the scatter plot of the estimated vs. measured LA values are presented in Fig 6, in which closeness of LA points to 1:1 line shows the high accuracy of this model.

#### ANN results

Different combinations of ANNs were trained and evaluated using 2019 data and the most accurate ANN models were selected based on the performance criteria. The selected ANN structures are presented in Table 6. Regarding the statistical criteria, the ANN model with LM training function, LS transfer function, and 21 neurons in the hidden layer (LM-LS-21), was the most accurate ANN model having R^2^ and RMSE values of 0.9969 and 0.3420, respectively, on the training data.

**Table 6:** The most accurate ANN models trained with 2019 data for estimation of LA based on leaf length and width.

Training function	Transfer function	Neurons in hidden layer	R^2^	RMSE
Bayesian regularization	Logarithm sigmoid	25	0.9966	0.3532
	Tangent sigmoid	22	0.9964	0.3695
**Levenberg-Marquardt**	**Logarithm sigmoid**	**21**	**0.9969**	**0.3420**
	Tangent sigmoid	17	0.9967	0.3568
Scaled conjugate gradient	Logarithm sigmoid	24	0.9957	0.4045
	Tangent sigmoid	21	0.9953	0.4195

**Fig 7:**
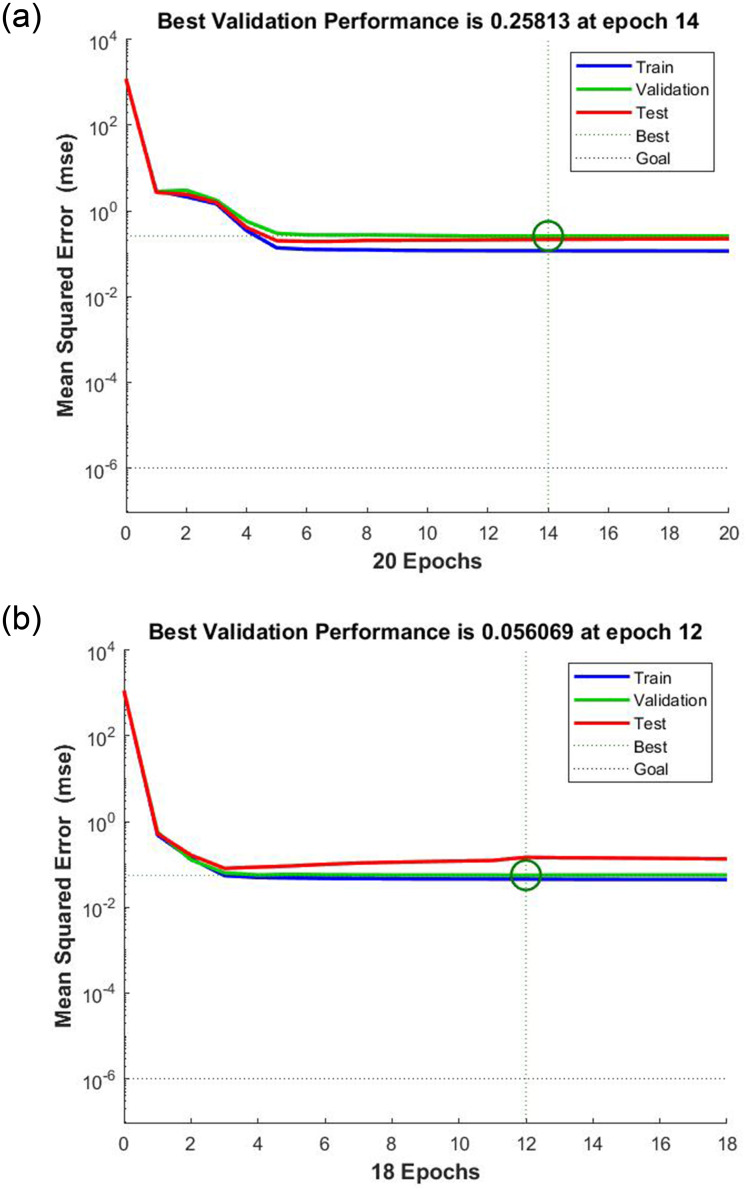
Variations of MSE value for training, testing and validation datasets during the training process of the optimized ANNs; a) the LM-LS-21 model for 2019 data, b) the LM-LS-27 model for 2021 data.

Fig 7a depicts the variations of the Mean Squared Error (MSE) values during the training process of the optimal ANN for training, validation and test datasets. It can be observed that the training was terminated by validation check at epoch 14 for the data of 2019. The least MSE value of this model on the validation data obtained 0.25813.

**Fig 8:**
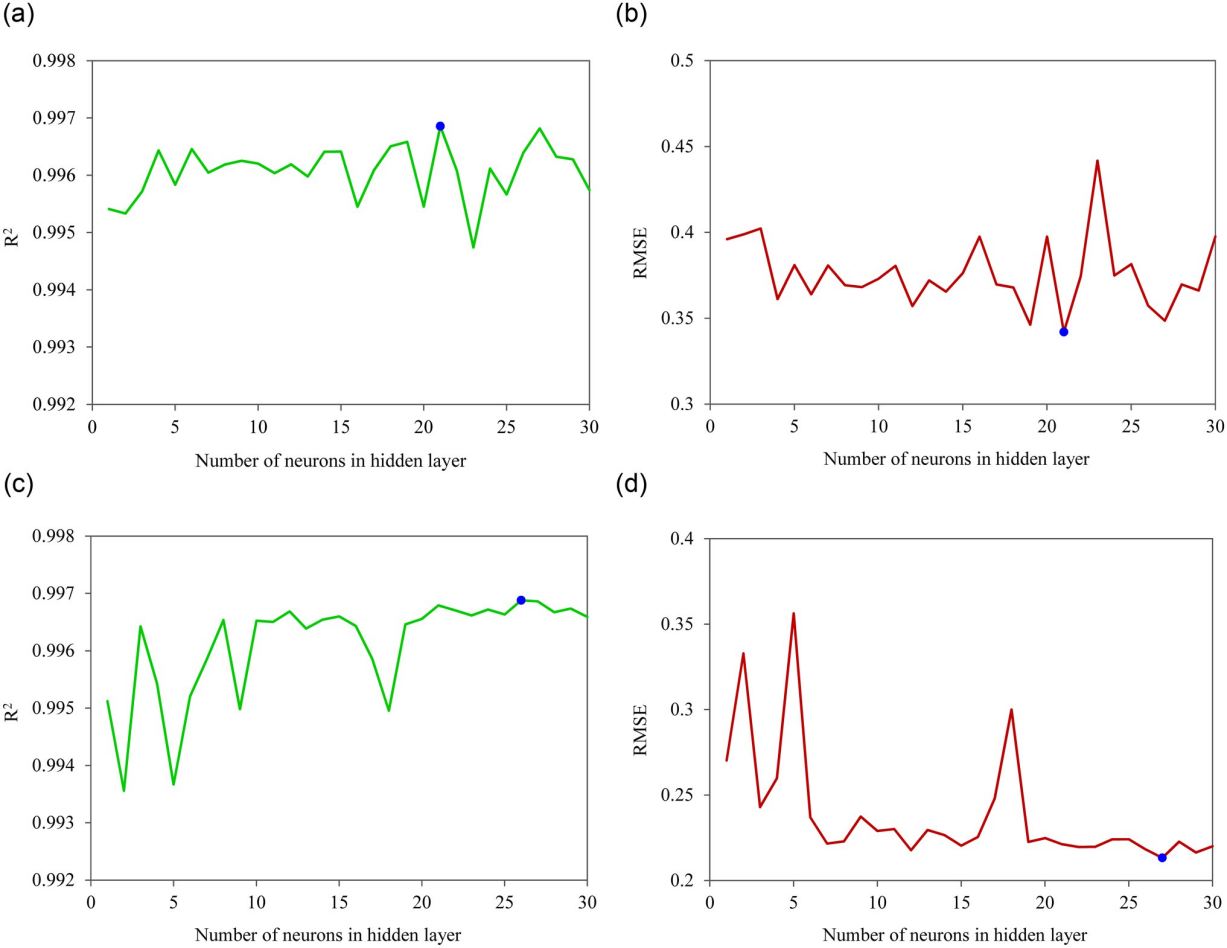
Performance criteria for ANN models with different numbers of neurons in the hidden layer, a) variation of the R^2^ value of the LM-LS-21 model for 2019 data, b) variation of the RMSE value of the LM-LS-21 model for 2019 data, c) variation of the R^2^ value of the LM-LS-27 model for 2021 data, d) variation of the RMSE value of the LM-LS-27 model for 2021 data.

In Fig 8, the effect of the number of neurons in the hidden layer on the performance values of the LM-LS arrangement is graphically presented. It can be observed that, for the 2019 data, the highest R^2^ and the smallest RMSE value of the ANN model were obtained when 21 neurons were used in the hidden layer (Fig 8a and 8b).

**Fig 9:**
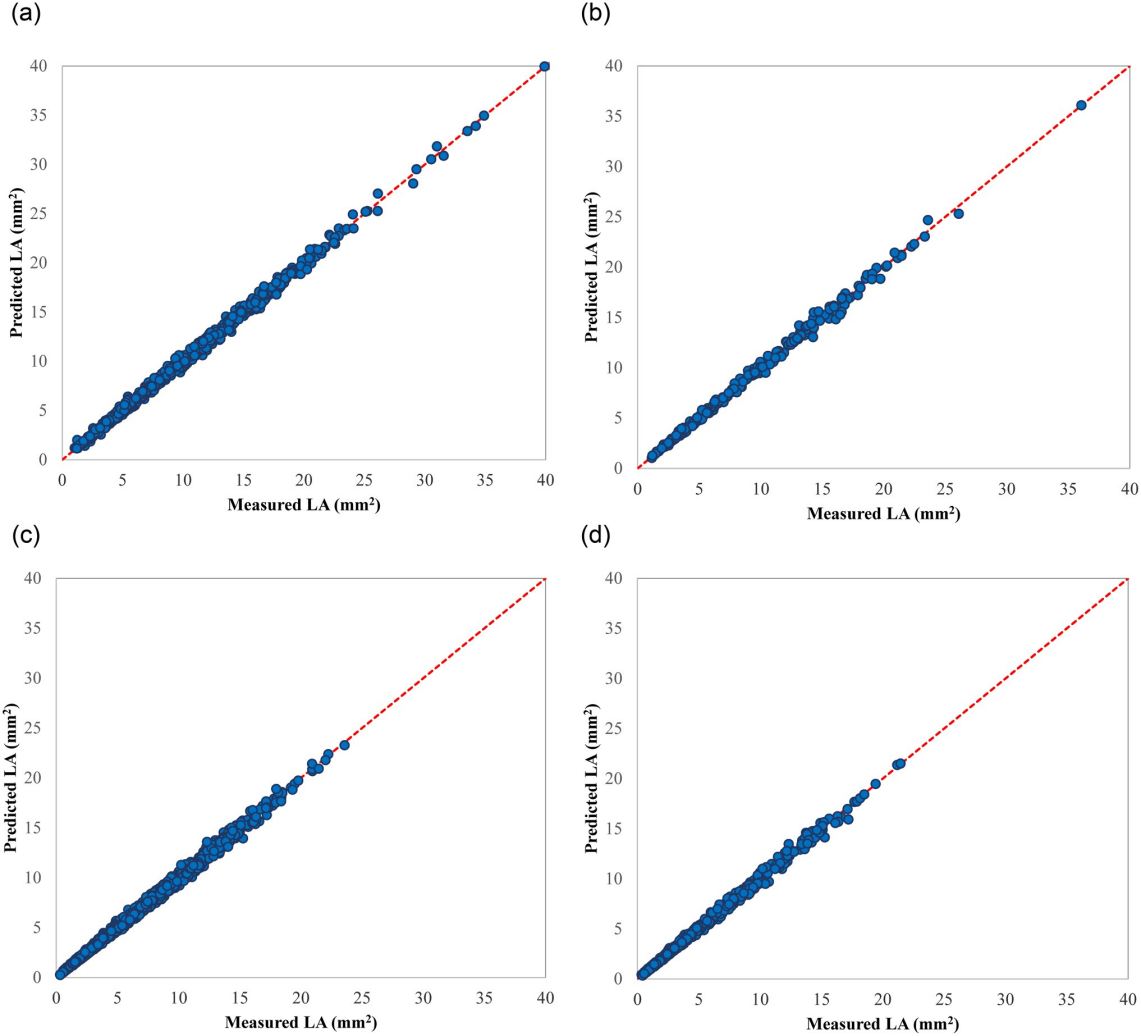
Scatter plots of the estimated LA values of selected ANN models v. the corresponding measured values; a) the 2019 data-driven ANN on the training dataset, b) the 2019 data-driven ANN on the test dataset, c) the 2021 data-driven ANN on the training dataset, and d) the 2021 data-driven ANN on the test dataset.

This optimize ANN model was evaluated on a separated test set of 2019 recordings which was not involved in the training process. The resulting R^2^ and RMSE measures were obtained 0.9966 and 0.3618, respectively. Fig 9a and 9b depict the graphical results of the ANN model on the training and test dataset. Closeness of the points to the one-to-one line (red line) both train and test datasets shows the high prediction ability of the ANN model.

The selected ANN model was also evaluated on the data of the 2021 data gathering experiments and the statistical values are presented in Table 7. The statistics in Table 7 prove the high ability of the ANN model trained with the data of one year to be used for LA estimation of plum genotypes even in future years.

**Table 7:** Evaluation results of the 2019 data-driven ANN model on the data collected in 2021 from three greengage plum genotypes and one myrobalan plum.

Genotype name	R^2^	RMSE
Gavali	0.9927	0.3433
Shahryari	0.9964	0.2365
Ghandi	0.9943	0.2289
Jangali	0.9930	0.2262

In order to include the information of all genotypes in the process of model building, to obtain a more comprehensive plum LA estimator model, the 2021 recorded characteristics of plum leaves were used to train ANNs. The performance criteria of the most appropriate ANN structures on the training set are given in Table 8. Table 8 shows that all the selected structures have a high capability to be used as a universal LA estimator. It can also be observed that the optimized comprehensive ANN model is comprised of 27 neurons in the hidden layer, LM training algorithm and LS activation function (LM-LS-27). This model gave an R^2^ of 0.9969 and an RMSE of 0.2131 on the training set. The training behavior of the selected ANN model is shown in Fig 7b. It can be seen that the training process of this model was stopped at epoch 12 by validation check and the best validation MSE reached to 0.056069 during the training. Furthermore, the changes of the R^2^ and RMSE values of the LM-LS combination by changing the number of neurons in the hidden layer is presented in Fig 8c and 8d, respectively, for 2021 data.

**Table 8:** The most accurate ANN models trained with 2021 data for estimation of LA based on leaf length and width.

Training function	Transfer function	Neurons in hidden layer	R^2^	RMSE
Bayesian regularization	Logarithm sigmoid	16	0.9968	0.2206
	Tangent sigmoid	14	0.9967	0.2224
**Levenberg -Marquardt**	**Logarithm sigmoid**	**27**	**0.9969**	**0.2131**
	Tangent sigmoid	20	0.9968	0.2156
Scaled conjugate gradient	Logarithm sigmoid	12	0.9953	0.2633
	Tangent sigmoid	19	0.9961	0.2430

The selected LM-LS-27 ANN model was tested on the independent data which was not used for the training, and found to be very promising, with R^2^ of 0.9967 and RMSE of 0.2267. High agreement between experimental and estimated LA values can be seen in Fig 9c and 9d.

### ANFIS results

Performance statistics of the top five ANFIS structures for LA estimation based on leaf L and W values are reported in Table 9. The highest accuracy was obtained by an ANFIS model trained using the hybrid optimization method, sigmoid input membership function, linear output membership function, and four membership functions for each input variable. This model resulted in R^2^ and RMSE values of 0.9971 and 0.3240, respectively, on the training dataset. This model yielded the R^2^ of 0.9967 and RMSE of 0.3435 on the test data.

**Table 9:** Performance criteria of the five most accurate ANFIS structures trained with 2019 data for LA estimation.

Optimization Method	Input Membership Function	Output Membership Function	Number of membership functions	R^2^	RMSE
**Hybrid**	**Sigmoid**	**Linear**	**4**	**0.9971**	**0.3240**
Hybrid	Gaussian	Linear	4	0.9967	0.3543
Hybrid	Sigmoid	Linear	3	0.9966	0.3596
Hybrid	Gaussian	Linear	3	0.9965	0.3605
Hybrid	Triangular	Linear	4	0.9965	0.3623

**Fig 10:**
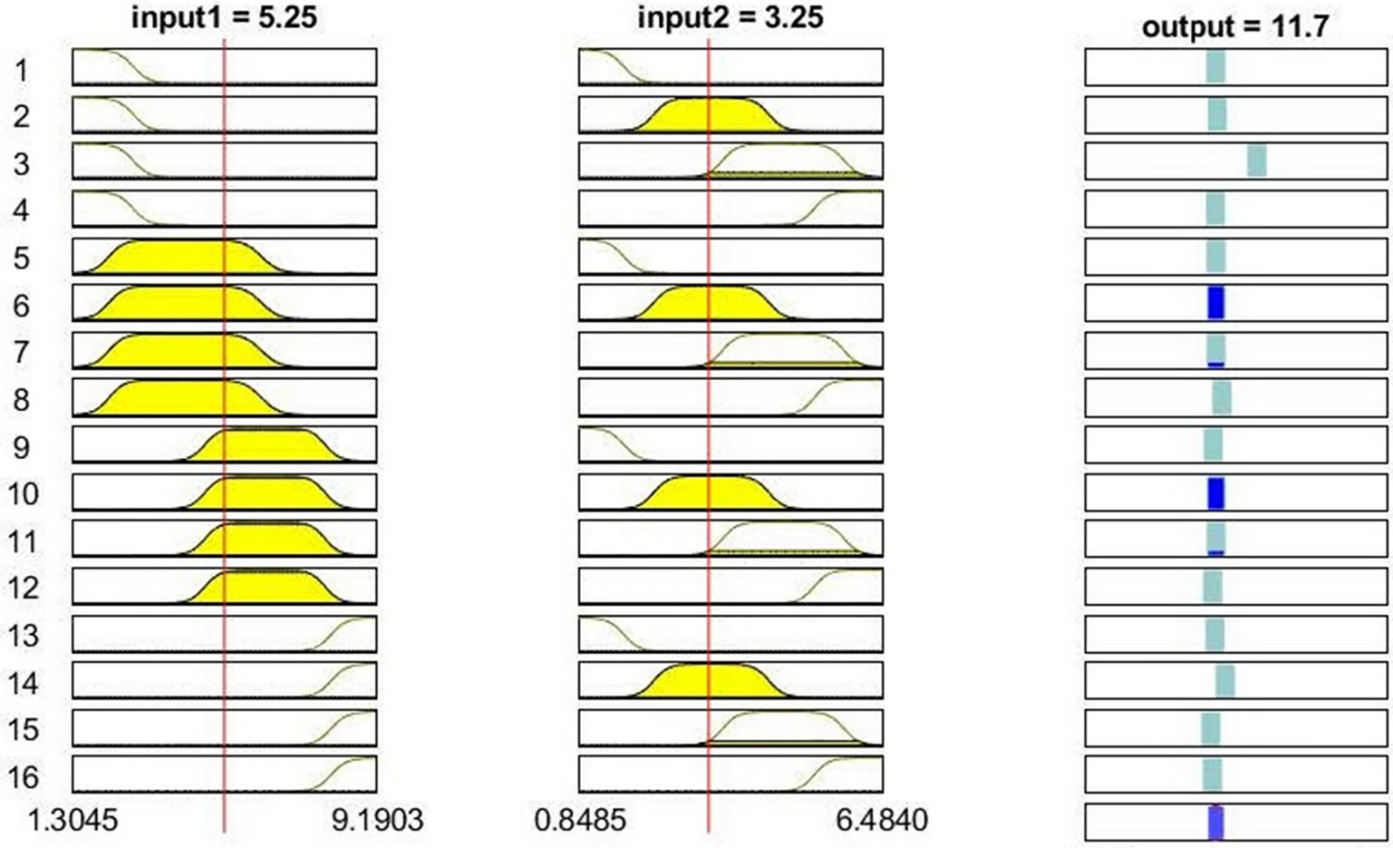
Fuzzy linguistic rules of optimized ANFIS model driven from 2019 data.

Linguistic rules of the most accurate ANFIS model are illustrated in Fig 10. Having four membership functions for each input variable, this ANFIS model generated 16 rules that are relating two input variables (leaf L and W) to the output (LA). As an example, it can be observed from Fig 10 that when the input 1 (leaf L) is 5.25 mm, and the input 2 (leaf W) is 3.25 mm, the final output (LA) will be calculated by the model as 11.7 mm^2^.

The most optimal 2019 data-driven ANFIS model was validated against the LA data from 2021 experiments to estimate the LA of four different plum genotypes. The corresponding R^2^ and RMSE values are available in Table 10. The data in Table 10 show the reliability of this ANFIS model to be used for the measurement of the plum LA future years.

**Table 10:** Evaluation results of the 2019 data-based trained ANFIS model on the data collected in 2021 from three greengage plum genotypes and one myrobalan plum.

Genotype name	R^2^	RMSE
Gavali	0.9942	0.2188
Shahryari	0.9971	0.2071
Ghandi	0.9965	0.1489
Jangali	0.9965	0.1024

**Fig 11:**
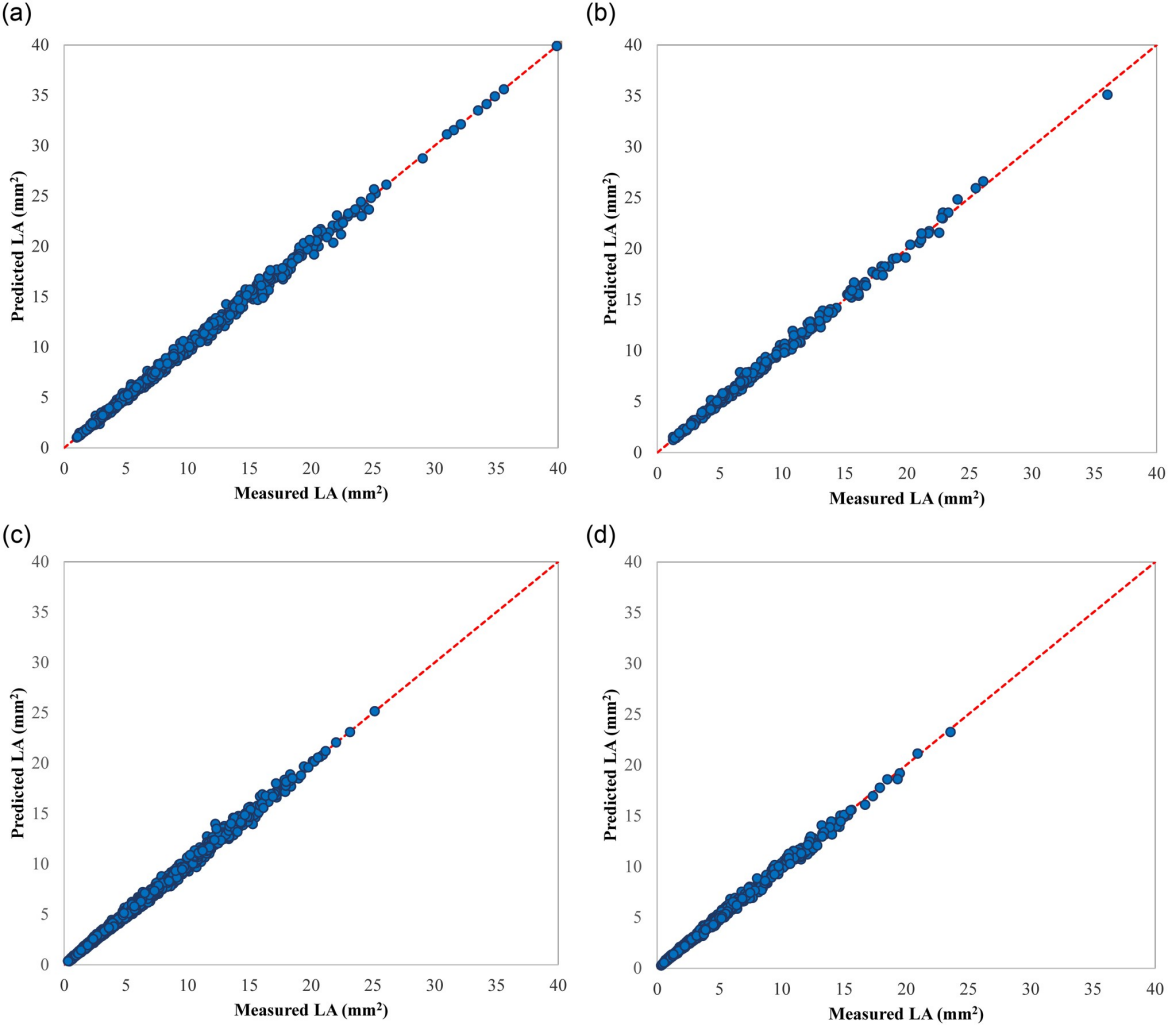
Scatters plot of measured vs. estimated LA values of selected ANFIS models; a) the 2019 data-driven ANFIS on the training dataset, b) the 2019 data-driven ANFIS on the test dataset, c) the 2021 data-driven ANFIS on the training dataset, and d) the 2021 data-driven ANFIS on the test dataset.

ANFIS was used to develop a single comprehensive LA estimator for all of the studied genotypes, and the performance values of the most accurate models are presented in Table 11. Same as that resulted from the analysis of 2019 data, the combination of the Hybrid optimization algorithm with sigmoid input membership function, linear output membership function, and four membership functions for each input variable, resulted in the most accurate model with R^2^ of 0.9971 and RMSE of 0.2101. The top-ranked model was validated using test data, and the obtained R^2^ and RMSE measures were 0.9964 and 0.2260, respectively. The ANFIS estimated values vs. the experimentally observed LA values for the training and testing dataset of the 2019 data-driven ANFIS are shown in Fig 11a and 11b, respectively. It can be seen that the data points are close to the ideal fitting line.

**Table 11:** Performance criteria of the five most accurate ANFIS structures trained with 2021 data for LA estimation.

Optimization Method	Input Membership Function	Output Membership Function	Number of membership functions	R^2^	RMSE
**Hybrid**	**Sigmoid**	**Linear**	**4**	**0.9971**	**0.2101**
Hybrid	Gaussian	Linear	4	0.9971	0.2105
Hybrid	Sigmoid	Linear	3	0.9970	0.2149
Hybrid	Triangular	Linear	4	0.9970	0.2162
Hybrid	Gaussian	Linear	3	0.9970	0.2164

It can be seen from Tables 9 and 11 that the ranking of the top five ANFIS models for 2021 data is almost similar to that of the 2019 model (there was only one difference in raking) showing the superiority of these structures to be used for developing ANFIS based plum LA prediction tool. All of the top accurate ANFIS models use the Hybrid optimization method and linear output membership function. Fig 11c and 11d depict, respectively, the distribution of the results of training and testing phases of the 2021 data-driven ANFIS, compared against the measured LA data. The scatter dots are lie very close to the perfect agreement line, showing the model’s high accuracy.

### SVR results

**Fig 12:**
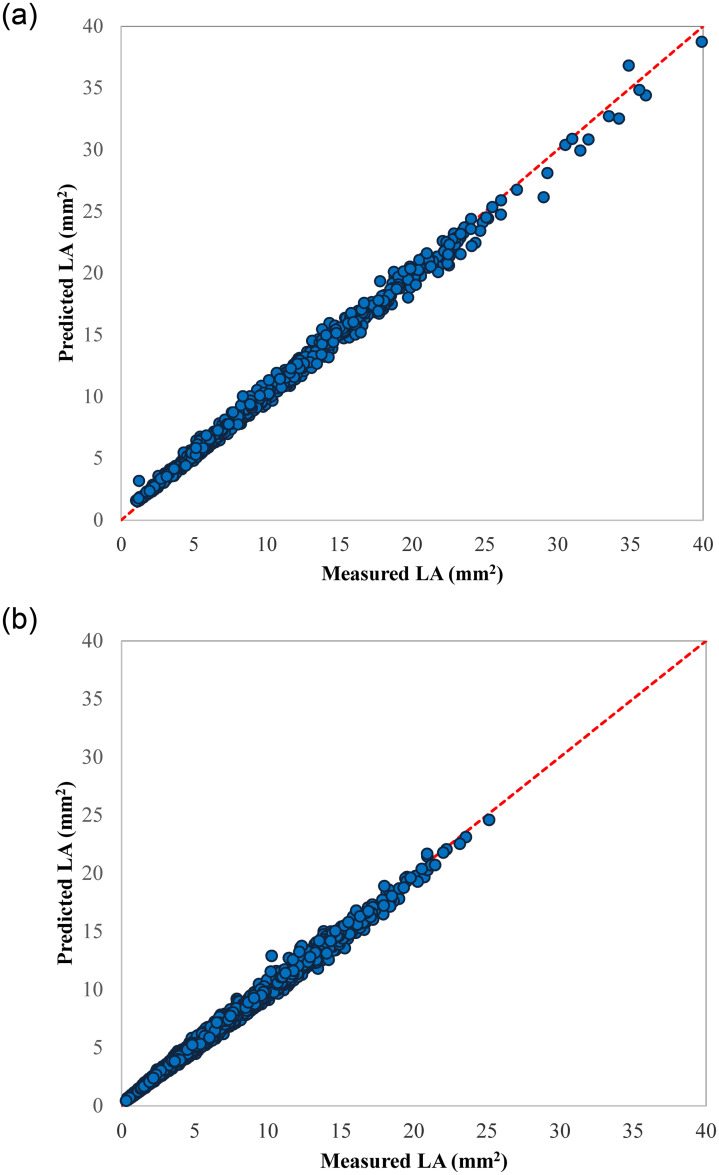
Scatter plots of estimated LA values by linear SVR model vs. the corresponding measured LA values, a) 2019 experiments, and d) 2021 experiments.

The performance results of SVR models with different kernel functions for LA estimation are provided in Table 12 for model development based on the data acquired in 2019 and 2021. The performance criteria in Table 12 depict that linear kernel SVR was the most accurate model among the evaluated SVRs. This model was able to predict LA based on leaf L and W with R^2^ of 0.9955 and RMSE of 0.4871, when it was used to model LA data of 2019 experiments. The graphical results of the linear SVR model of 2019 data can be seen in Fig 12a which shows almost high concordance of the SVR estimated LA values with the measured LA values.

**Table 12:** Performance criteria of SVR models with different kernels for data of years 2019 and 2021.

Kernel type	2019 data		2021 data	
	R^2^	RMSE	R^2^	RMSE
Linear	**0.9955**	**0.4871**	**0.9950**	**0.2959**
2 dimensional	0.7278	3.1519	0.7790	1.8055
3 dimensional	0.7889	2.8111	0.8280	1.6003
RBF	0.9967	0.3568	0.9863	0.5029

The SVR model that was derived from 2019 data, was validated using LA data of 2021 experiments, and the obtained R^2^ and RMSE values on four plum genotypes are presented in Table 13. The highly desired criteria in Table 13 confirm the reliability of the SVR model as an excellent LA estimator with R^2^ values of more than 0.992.

**Table 13:** Evaluation results of the 2019 data-based trained SVR model on the data collected in 2021 from three greengage plum genotypes and one myrobalan plum.

Genotype name	R^2^	RMSE
Gavali	0.9926	0.3360
Shahryari	0.9971	0.4834
Ghandi	0.9936	0.5492
Jangali	0.9941	0.1439

In another effort, the SVR models were used to develop a universal LA predictor model based on the 2021 data. The performance values of different evaluated SVRs in this section are also available in Table 12. In this case, the linear kernel SVR was the most accurate model giving the R^2^ and RMSE values of 0.9950 and 0.2959, respectively. The closeness of the scattered points around the 1:1 line proves the robustness of the linear SVR model (Fig 12a and 12b).

## Discussion

Four computational modeling techniques, including LRM, SVR, ANN, and ANFIS, were used and compared to estimate the plum’s LA. Two distinct species were investigated, including three greengage genotypes and one myrobalan plum. The evaluation results of the image processing algorithm demonstrated that the dimensional leaf properties could be measured with high accuracy using image analysis techniques. As a result of a large number of leaves collected, image analysis was used to extract the desired data for model development. The capability of the aforementioned modeling techniques was evaluated using two different sets of data from the years 2019 and 2021.

In terms of performance criteria, all of the approaches above successfully estimated the LA from leaf L and W values. This is primarily due to the high intrinsic correlation between the W and L values of the resulting plum leaves and their area. Additionally, the high accuracy of the LRM method, compared to the linear SVR method, demonstrates a strong linear correlation between perpendicular dimensions and the area of plum leaves. The R^2^ and RMSE values of linear kernel SVR for LA estimation were 0.9955 and 0.4871, respectively when fitted to 2019 data. Concerning LRM, the LA = 0.007 + 0.687 L×W model was able to estimate LA with an R^2^ = 0.9955 and an RMSE = 0.4037 using the product of leaf L and W. There is a wide range of reported accuracy values for LA estimation using the product of L×W, ranging from 0.68 for citrus [50] to 0.980 for loquat [9].

Using data from 2019, the ANN and ANFIS models demonstrated superior performance criteria during the model development process. R^2^ values for ANN and ANFIS models were 0.9969 and 0.9971, respectively, while RMSE values for ANN and ANFIS were 0.3420 and 0.3240, respectively. Examining these values in aggregate demonstrates that neurocomputing approaches outperform classical modeling techniques such as LRM and SVR.

Küçükönder and Boyaci [31] showed that the ANN method outperformed the regression method for estimating the LA of tomato plants. Asriani [35] reported a considerable performance value of 99.99% in predicting the LA of seven plant species based on leaf L and W. These species included palm leaves, maize, thatch, chili, pepper, betel, and kale. Azeem and Javed [30] conducted a comparative study and demonstrated the superiority of the ANN (R2 = 0.96) over the mathematical method (R^2^ = 0.94) for estimating the LA of rabbits paw weed. Similarly, reports on the LA estimation of durian [11], *Ormosia paraensis* [51], pear cultivars [52], red chief apple [53], and bell pepper [54] have been published. Moreover, Amiri and Shabani [55] described the ANFIS method as accurate (R^2^ = 0.997) for LA prediction.

The most accurate models developed using the 2019 dataset were validated using new sets of data from four distinct plum genotypes collected in 2021. As a result of the significant accuracy (R^2^ > 0.992), all methods can be considered highly appropriate for non-destructive plum LA estimation.

In another research phase, a universal model was developed using data from 2021, and it was determined that the ANFIS method was again the most accurate model, with an R2 = 0.9971 and an RMSE = 0.2101. ANN was ranked first (R^2^ = 0.9969; RMSE = 0.2131), followed by LRM (R^2^ = 0.9950; RMSE = 0.2723), and Linear SVR (R^2^ = 0.9950; RMSE = 0.2959). These results indicate that ANFIS outperforms the ANN, SVR, and LRM methods for estimating the LA from L and W values.

The ANFIS model was the most accurate LA estimator in the study’s performance criteria. This finding is consistent with Sabouri and Sajadi’s [38] findings, who reported that the ANFIS was more accurate than ANN and regression methods for estimating the LA of wheat and triticale leaves. Comparison of the selected models derived from each genotype with the final model (achieved by pooling all genotypes data) proved that the developed models accommodated the effect of changes in leaf shape between genotypes and could be used for other genotypes of plum with considerable accuracy. In order to achieve a reliable and accurate model, the sample size is too important. In order to develop a prediction model, the sample size must be large enough to ensure stable coefficients. The larger the sample size, the more reliable results [56]. Using an inadequate sample size, the model may not predict well and be acceptable for future subjects [57]. In this research, a large sample size for each genotype was used, and stable performance coefficients were obtained. Given the evidence, it is possible to conclude that the final universal model, which can accurately predict the LA of different plum genotypes, can be used as a reliable LA estimator model for plum genotypes.

## Conclusion

Plant LA estimation is a critical indicator of plant growth and health. The development of precise, reliable, non-destructive, and non-invasive techniques for LA monitoring continues to be a focus of research. Conventionally, mathematical modeling has been used to estimate the LA using leaf L and W values non-destructively. In this study, three machine learning techniques of SVR, ANN, and ANFIS were compared with LRM method to determine the most powerful algorithm for estimating LA based on leaf L and W. The experiments were performed across two years, 2019 and 2021, and used a large sample size to ensure reliable results. Due to the high accuracy of the image processing method in extracting the size parameters, it was employed to collect dimensional data from a total number of 5548 leaves. The results indicated that methods based on artificial intelligence are more accurate than those based on mathematical methods. For both years, the ANFIS model was the most accurate LA estimator. The R^2^ and RMSE values for the ANFIS, ANN, LRM, and SVR methods were 0.9971 and 0.3240; 0.9969 and 0.3420; 0.9955 and 0.4037; and 0.9955 and 0.4871, respectively, based on their performance rank on data from 2019. For 2021 data, a similar performance rank order was observed, with R^2^ and RMSE values of 0.9971 and 0.2101 for ANFIS; 0.9969 and 0.2131 for ANN; 0.9950 and 0.2723 for LRM; and 0.9950 and 0.2959 for SVR. Furthermore, evaluation of the developed ANFIS model using selected 2019 and 2021 data for various plum genotypes demonstrated this approach’s broad applicability for estimating LA in the future. According to the findings of this study, it is recommended that artificial intelligence be used to estimate LA instead of the regression method. Moreover, it is very encouraging that the ANFIS modeling method accurately estimated the LA from image extracted features. According to the findings of this study, an accurate, portable, and simple-to-use of non-destructive LA estimation system is conceivably close to being reached due to the high potential of artificial intelligence in the development of PC and phone applications.

## Supporting information

S1 Data (XLSX)

S1 Table Experimental leaf length, width and area values of Gavali greengage genotype in 2019. (XLSX)

S2 Table Experimental leaf length, width and area values of Gavali greengage genotype in 2021. (XLSX)

S3 Table Experimental leaf length, width and area values of Ghandi greengage genotype in 2021. (XLSX)

S4 Table Experimental leaf length, width and area values of Shahryari greengage genotype in 2021. (XLSX)

S5 Table Experimental leaf length, width and area values of Jangali myrobalan plum in 2021. (XLSX)
